# An N-Shaped Lightweight Network with a Feature Pyramid and Hybrid Attention for Brain Tumor Segmentation

**DOI:** 10.3390/e26020166

**Published:** 2024-02-15

**Authors:** Mengxian Chi, Hong An, Xu Jin, Zhenguo Nie

**Affiliations:** 1 School of Computer Science and Technology, University of Science and Technology of China, Hefei 230026, China; 2 Department of Mechanical Engineering, Tsinghua University, Beijing 100084, China; 3 State Key Laboratory of Tribology in Advanced Equipment, Tsinghua University, Beijing 100084, China; 4 Beijing Key Laboratory of Precision/Ultra-Precision Manufacturing Equipments and Control, Tsinghua University, Beijing 100084, China

**Keywords:** brain tumor segmentation, CNNs, feature pyramid, lightweight model, hybrid attention

## Abstract

Brain tumor segmentation using neural networks presents challenges in accurately capturing diverse tumor shapes and sizes while maintaining real-time performance. Additionally, addressing class imbalance is crucial for achieving accurate clinical results. To tackle these issues, this study proposes a novel N-shaped lightweight network that combines multiple feature pyramid paths and U-Net architectures. Furthermore, we ingeniously integrate hybrid attention mechanisms into various locations of depth-wise separable convolution module to improve efficiency, with channel attention found to be the most effective for skip connections in the proposed network. Moreover, we introduce a combination loss function that incorporates a newly designed weighted cross-entropy loss and dice loss to effectively tackle the issue of class imbalance. Extensive experiments are conducted on four publicly available datasets, i.e., UCSF-PDGM, BraTS 2021, BraTS 2019, and MSD Task 01 to evaluate the performance of different methods. The results demonstrate that the proposed network achieves superior segmentation accuracy compared to state-of-the-art methods. The proposed network not only improves the overall segmentation performance but also provides a favorable computational efficiency, making it a promising approach for clinical applications.

## 1. Introduction

Brain tumor segmentation plays a crucial role in the diagnosis, treatment planning, and monitoring of brain tumors [1]. Accurate segmentation of brain tumor regions from multi-sequence magnetic resonance imaging (MRI) data is of paramount importance for precise tumor analysis and subsequent clinical decision making [2]. The ability to delineate tumor boundaries in MRI scans enables radiologists and clinicians to assess tumor size, location, and heterogeneity, facilitating treatment planning and evaluating treatment response [3]. Traditional manual segmentation methods are time-consuming, subjective, and prone to inter-observer variability [4]. Therefore, the automatic segmentation algorithm has received widespread attention as an alternative solution. For instance, the self-organizing map (SOM) [5] is an unsupervised exploratory data analysis tool that leverages principles of vector quantization and similarity measurement to automatically partition images into self-similar regions or clusters. Segmentation methods based on SOM have demonstrated the ability to distinguish high-level and low-level features of tumors, edema, necrosis, cerebrospinal fluid, and healthy tissue [6,7].

Furthermore, with the rapid advancements in deep learning techniques, particularly convolutional neural networks (CNNs), there has been a paradigm shift in the field of brain tumor segmentation [8,9]. Moreover, the application of the U-Net model in brain tumor segmentation has led to significant strides in this field [10]. The remarkable progress achieved by the U-Net model and its variants can be attributed to its unique architectural design, which addresses several challenges specific to brain tumor segmentation [11,12]. Firstly, the U-Net model adopts a fully convolutional network architecture, eliminating the need for fully connected layers typically found in traditional CNNs. This design choice enables the U-Net model to handle input images of arbitrary sizes, making it well-suited for the analysis of medical images with varying dimensions. Additionally, the U-Net model incorporates transpose convolution, also known as deconvolution or up-sampling, which allows up-sampling of the learned features to the original input size. This capability is particularly advantageous in brain tumor segmentation, as it enables the U-Net model to generate segmentation maps with the same resolution as the input images, preserving fine-grained details necessary for accurate tumor delineation. By combining these architectural features, the U-Net model exhibits superior performance in capturing contextual information, localizing tumor boundaries, and accurately segmenting brain tumor regions [13].

However, despite its remarkable performance, the U-Net model does have certain limitations that should be taken into consideration. Firstly, the U-Net model relies on an encoder–decoder structure, where the encoder captures contextual information while the decoder performs up-sampling for precise localization [14]. Nevertheless, this design may result in the loss of fine-grained details during the down-sampling and up-sampling processes, which can affect the accuracy of tumor segmentation, especially for small or subtle tumor regions. Additionally, an important consideration when using the U-Net model is its large model size [15]. U-Net architecture typically requires a significant number of parameters and memory to accommodate the expansive feature maps and deep network structure. This large model size can pose challenges in terms of deployment and practical usage, especially in resource-constrained environments such as mobile devices or real-time clinical applications. Furthermore, the U-Net model may struggle with handling class imbalance issues, which are commonly encountered in brain tumor segmentation tasks, where the tumor regions typically occupy a small portion of the overall image [16]. Class imbalance can lead to biased training and produce suboptimal segmentation results.

In this work, our focus lies in proposing a novel neural network architecture for multi-sequence MRI brain tumor image segmentation to address the aforementioned challenges faced by the U-Net model and further improve the accuracy and efficiency of tumor segmentation.

To address the issue of fine-grained information loss inherent in the encoder-decoder structure of U-Net, we introduce the N-shaped neural network, which uniquely combines the core concepts of multi-path CNNs, Feature Pyramids [17], and the U-Net architecture [18]. Notably, the proposed N-shaped neural network incorporates the novel Multiple Feature Pyramid (MFP) paths as a multi-path CNN component. These MFP paths extract features at multiple scales from brain tumor images and seamlessly transmit them to the encoder. By integrating the MFP paths, the N-shaped neural network adeptly handles variations in tumor size, shape, and appearance, thereby showcasing improved segmentation performance. Consequently, the N-shaped neural network effectively harnesses the strengths of both multi-path CNNs and U-Net architectures, enabling it to capture diverse contextual information while accurately localizing and preserving fine-grained details.

In addition, to effectively reduce the model’s parameter size and computational complexity while maintaining performance, we propose a novel lightweight convolution module that integrates hybrid attention mechanisms. This integration has not been explored before, making it a unique contribution to the field. The commonly used deep separable convolution (DSC) module [19] often faces performance degradation. To overcome this limitation, we investigate the insertion of a spatial attention module [20] after the depth-wise convolution and a channel attention module [21] after point-wise convolution. Remarkably, this configuration significantly enhances the performance of the deep separable convolution module. Consequently, we term this module configuration, consisting of the sequence of Depth-wise convolution, Spatial attention module, Point-wise convolution, and Channel attention module, as the DSPC module. Additionally, we explore the application of different attention mechanisms in the horizontal skip connections of our proposed model. Through meticulous experimentation, we discover that the channel attention mechanism yields the most promising results.

Finally, to further effectively address the challenge of tumor region imbalance in brain tumor segmentation, we employ a combo loss function that combines an improved weighted cross-entropy loss [22] and Dice loss [23] for supervised training. The weighted cross-entropy loss assigns higher weights to the minority class during training. We develop a unique weighting algorithm that utilizes sub-region-based weights instead of label-based weights. This innovative approach enables the model to be more sensitive to the smallest yet crucial enhancing tumor (ET) region, thus improving segmentation accuracy. Furthermore, Dice loss measures the overlap between the predicted and ground truth segmentation masks, providing a comprehensive evaluation of segmentation performance. By leveraging the strengths of both loss functions, our approach promotes accurate segmentation of brain tumor regions.

Our contributions are summarized as follows:

- To the best of our knowledge, this is the first work to propose the integration of the Multi-path CNN ideology, Feature Pyramid structure, and U-Net architecture into the novel N-shaped neural network structure, accompanied by the introduction of the MFP pathway module. This integration enables the extraction of multi-scale features from brain tumor images, enhancing the model’s capability for comprehensive analysis.
- N-LiNet utilizes the DSPC lightweight module as the foundational building block and integrates channel attention mechanisms in skip connections. This innovative approach, which explores the integration of attention mechanisms in different locations within the DSC module, is a novel contribution to the field. The hybrid module not only reduces the computational resources required by the model, but also improves its segmentation performance. Moreover, it provides valuable insights into the varying sensitivity of different modules within deep separable convolutions to different features, opening avenues for further research.
- To tackle the issue of class imbalance in brain tumor segmentation, we propose a novel combo loss function that combines the improved weighted cross-entropy loss and Dice loss. We introduce a sub-region-based weighting algorithm specifically designed for brain tumor segmentation, which, to our knowledge, has not been previously explored. By assigning higher weights to tumor regions, our approach provides a unique solution to tackle the problem of class imbalance, enhancing the segmentation accuracy and contributing to the field of brain tumor analysis.

By comparing the proposed N-shaped Lightweight neural Network (N-LiNet) architecture with several state-of-the-art segmentation models [24,25,26,27,28,29,30,31,32] on multiple publicly available brain tumor segmentation datasets (UCSF-PDGM [33], BraTS [34], and MSD [35]), N-LiNet achieves superior segmentation metrics with lesser parameter size and computational complexity. The performance of N-LiNet highlights its effectiveness in accurately segmenting brain tumors while minimizing resource requirements, making it a promising solution for practical applications in medical image analysis.

The subsequent sections of this paper are organized as follows. Section 2 provides an overview of the related work in the field of brain tumor segmentation. Section 3 presents the methodology employed in this study, focusing on the proposed N-LiNet architecture and its components. In Section 4, the datasets and evaluation metrics utilized for the experiments are introduced. Section 5 presents the experimental results and analysis, discussing the segmentation performance of N-LiNet compared to state-of-the-art methods on the selected datasets. Finally, Section 6 concludes the paper by summarizing the contributions, discussing the limitations, and outlining potential directions for future research in the field of brain tumor segmentation.

## 2. Related Work

### 2.1. Attention Mechanisms in CNNs

**Figure 1:**
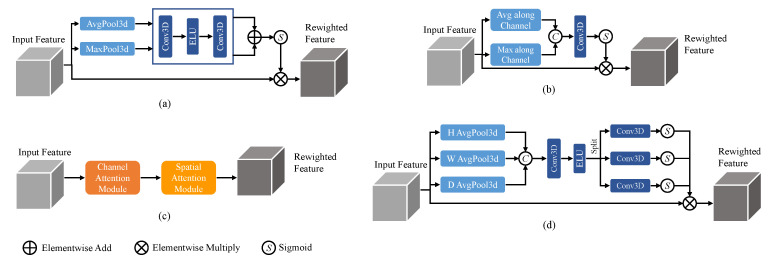
Attention mechanisms with (**a**) channel attention, (**b**) spatial attention, (**c**) CBAM, and (**d**) coordinate attention.

The channel attention mechanism [21] focuses on enhancing important channel information while suppressing less relevant channels, achieved by computing attention weights along the channel dimension. It typically involves global average pooling and a multi-layer perceptron (MLP) to generate attention weights, which are then used to weight the original feature maps. On the other hand, the spatial attention mechanism [20] aims to highlight important spatial locations and suppress unimportant ones. It can be implemented through convolutional operations with different kernel sizes or using self-attention mechanisms to compute attention weights between spatial positions. These attention mechanisms, when combined, form the Convolutional Block Attention Module (CBAM) [36], which integrates both channel and spatial attention. Additionally, a recently proposed attention mechanism called coordinate attention [37] focuses on modeling the relationships between different positions in the feature maps by incorporating coordinate information. It utilizes an MLP to process the coordinate information and generates position weights, which are then multiplied with the original feature maps. The 3D structures of these commonly used attention mechanisms are illustrated in Figure 1. While these attention mechanisms enhance the modeling capability of CNNs by capturing important channel, spatial, and positional information, they also introduce additional computational overhead.

### 2.2. Single-Path and Multi-Path CNNs for Brain Tumor Segmentation

CNNs have shown great promise in achieving accurate and efficient segmentation results, revolutionizing the way brain tumors are analyzed and diagnosed due to their ability to automatically learn discriminating features from input data [38]. Initially, single-path CNNs were employed, where a single data processing stream was utilized [39]. These networks, which take multi-modal brain tumor MRI scans as input, sequentially pass the data through a combination of convolutional layers, pooling layers, and non-linear activation layers, ultimately performing segmentation using a classifier at the end of the model. Single-path CNNs are characterized by their simplicity in structure and shallow hierarchy, but their segmentation performance may be sub-optimal. As brain tumor images are inherently complex and diverse, relying solely on a single processing path may limit the network’s ability to capture and represent the intricate details present in different modalities.

To address this limitation, multi-path CNNs have been introduced, featuring multiple parallel convolutional pathways [40]. This architecture allows for the processing of input information at multiple scales, providing a larger receptive field and the potential for enhanced segmentation accuracy. However, it is worth noting that multi-path CNNs tend to exhibit a higher level of complexity and require a larger model size to accommodate the increased number of pathways. Furthermore, an inherent challenge arises from the uneven distribution of tumor regions, where certain tumor areas may exhibit varying sizes and proportions compared to others.

To tackle this class imbalance issue, cascaded CNNs have been proposed as a potential solution [41]. By cascading multiple network models, each designed to segment a specific region of interest, cascaded CNNs enable the transformation of the multi-region tumor segmentation problem into a series of binary segmentation tasks. One of the key advantages of cascaded CNNs is their ability to consider the unique relationships between sub-regions when predicting subsequent segmentation tasks. This can be particularly beneficial in minimizing false positives, as each network operates on regions extracted from the output of the previous network. However, an important point to consider is that cascaded CNNs, in contrast to single-path and multi-path CNNs, are not end-to-end and require additional time for training and testing due to the sequential nature of the cascaded segmentation process.

### 2.3. The U-Net and Its Variants for Brain Tumor Segmentation

The U-Net architecture consists of an encoder–decoder structure coupled with skip connections [10]. The encoder path incorporates a series of convolutional and pooling layers to progressively extract hierarchical features and reduce spatial resolution. The decoder path utilizes up-sampling and transposed convolutional layers to recover the spatial information and generate segmentation maps. Skip connections connect the corresponding encoder and decoder layers, allowing for the model to fuse low-level and high-level features. This design enables U-Net to capture both local and global contextual information, facilitating the accurate delineation of tumor boundaries.

Initially, the research focused on 2D segmentation networks operating within individual 2D image planes. U-Net [12] has demonstrated its efficacy in capturing tumor boundaries and distinguishing tumor regions from healthy brain tissue. U-Net++ [25] extends the U-Net architecture by incorporating nested and dense skip pathways, enabling the capture of multi-scale contextual information for precise brain tumor segmentation. SegResNet [26] combines U-Net architecture with the residual network (ResNet) to enhance feature representation and segmentation performance, effectively capturing both local and global contextual information. To further improve feature representation, DynU-Net [27] integrates a dynamic routing algorithm inspired by capsule networks into the U-Net architecture, enabling the capture of hierarchical relationships among different tumor regions. MS-Net [31] is a medical image segmentation technique based on a codec structure composed of a Multi-Scale Attention Module (MSAM) and a Stacked Feature Pyramid Module (SFPM). MSAM dynamically adjusts the receptive fields to capture different levels of context details, while SFPM adaptively increases the weight of the features of interest to focus the network’s attention on the target region. Fusion factor [42] is introduced to control the amount of information transferred from deep to shallow layers in Feature Pyramid Networks (FPN) for tiny object detection. The paper explores how to estimate the effective value of the fusion factor for a specific dataset by statistical methods. However, these 2D networks may disregard the crucial depth information inherent in the MRI images, consequently impeding their ability to comprehensively utilize the rich local and global contextual information available.

Therefore, 3D U-Net [43] was developed to extend the U-Net framework for processing volumetric data, enabling the segmentation of brain tumors in 3D medical images. By considering spatial dependencies along the three dimensions, SCAR U-Net [44] improves the accuracy of tumor segmentation in volumetric scans. V-Net [45] is another extension of U-Net that incorporates a volumetric residual learning framework. It leverages 3D convolutional neural networks and residual connections to capture fine-grained details in volumetric data. The evolution of mainstream 2D segmentation networks into their 3D counterparts has resulted in significant improvements in brain tumor segmentation performance [46]. DSTGAN [47] presents a spatiotemporal generative adversarial learning approach for segmentation and quantification of myocardial infarction without contrast agents. The approach utilizes a generator and a discriminator module, which consist of three seamlessly connected networks to extract the morphological and motion abnormalities of the left ventricle, learn the complementarity between segmentation and quantification tasks, and leverage adversarial learning to enhance the accuracy of estimation. However, it remains crucial to strike a balance between model complexity and computational feasibility, considering the practical constraints and available computational resources.

Simultaneously, the transformer [48] architecture has gained significant popularity in natural language processing (NLP) and has found applications in medical image analysis [49,50]. Initially developed for sequence modeling tasks, transformers have showcased their ability to capture long-range dependencies and capture contextual information effectively. Building upon this success, researchers have extended transformers to medical image analysis, leading to the emergence of models. UNETR [30] combines the transformer architecture with the U-Net framework, enabling the modeling of long-range dependencies and achieving state-of-the-art performance in brain tumor segmentation. Similarly, SwinUNETR [28] integrates the Swin Transformer, a hierarchical vision transformer, with the U-Net framework, effectively capturing global and local dependencies with reduced computational complexity. nnFormer [32] is a novel approach using a 3D transformer to segment medical images based on interleaved convolution and self-attention operations. It introduces local and global volume-based self-attention to learn volume representations and outperforms previous transformer-based methods on three public datasets. SeMask [51] proposes a semantically masked transformer network for semantic segmentation of images. The network leverages an additional semantic layer to incorporate semantic information about the image, which improves the performance of the pre-trained transformer backbone. However, these transformer-based U-Net models face challenges such as increased model size, longer training time, and higher computational requirements, which can limit their practicality in real-world applications.

## 3. Methods

In this section, we commence by presenting a succinct overview of the architecture of the proposed N-shaped Lightweight Network (N-LiNet). Subsequently, we undertake an in-depth exploration of its constituent components, including the Multiple Feature Pyramid (MFP) paths, the sequence of Depth-wise convolution, Spatial attention, Point-wise convolution, and Channel attention (DSPC) module, and the integration of Channel Attention in the Horizontal skip connections (HCA). Afterward, we expound upon the design of a novel combo loss function.

### 3.1. Architecture Overview of the Proposed Network

**Figure 2:**
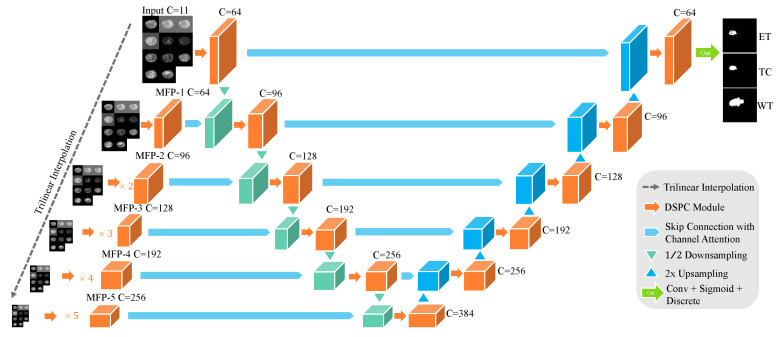
N-LiNet Architecture Overview.

The overall structure of N-LiNet, illustrated in Figure 2, consists of three primary components. On the left-hand side, the MFP paths are depicted, which encompass a Feature Pyramid pathway comprised of multiple CNNs. In the middle, the encoder component is situated, while on the right-hand side, the decoder component is located. Horizontal skip connections are utilized to establish interconnections among the three constituent parts. In addition to the aforementioned architectural components, we employ wider channels and deeper sampling levels in the design of N-LiNet to effectively handle the complexity and variability inherent in multi-sequence MRI brain tumor images.

After traversing the MFP paths, the feature maps are extracted and forwarded to their corresponding encoder modules. These feature maps are subsequently concatenated with the down-sampled feature maps acquired from the encoder path. Similarly, the features extracted through the encoder path are propagated to their corresponding decoder modules and then concatenated with the up-sampled features generated within the decoder path. Ultimately, the model reconstructs the segmented output with a resolution equivalent to that of the input images.

MFP paths serve as a feature extraction mechanism, leveraging a set of CNNs to produce a feature pyramid that captures multi-scale information. The encoder component further processes the extracted features, encoding them into a concise representation that retains significant semantic information. Subsequently, the decoder component utilizes the encoded features to reconstruct the desired output. Notably, the presence of horizontal skip connections facilitates the seamless flow of information across the various components, allowing for the fusion of high-level and low-level features and promoting effective information propagation throughout the network.

In summary, Algorithm 1 represents the overall algorithm flow design of N-LiNet.
**Algorithm 1** N-LiNet: A novel deep learning model for multi-sequence MRI brain tumor segmentation**Require:** Input Feature Maps: $F_{input}\left[B,C,H,W,D\right]$ *B: Batch size, C: Channel (MRI sequences), H: Height, W: Width, D: Depth*
      Ground Truth Labels: Y[B,C,H,W,D] *C: Classes*
      Initialized Weight Dictionary: $\Theta \{\theta_{input},\theta_{output},\ldots \}$ **Ensure:** Output Predictions: P[B,C,H,W,D]
1:**Module**    Input Layer2:**input **$F_{input}$3:$F^{\prime }\leftarrow$ GroupNorm(Conv($\theta_{input},F$))4:$F_{0}\leftarrow$ ELU(Add($F,F^{\prime }$))5:**return** $F_{0}$6: 7:**Module**    MFP    Multiple Feature Pyramid8:**input **$F_{input}$9:**for **i=1,...,5**do**10:   $F_{i}^{MFP}\leftarrow$ MFP-*i*($\theta_{i}^{MFP},F_{input}$)    {See Algorithm 2 for details}11:**end for**12:**return **$list[F_{i}^{MFP}]$13: 14:**Module**    ENC    Encoder15:**input **$F_{0},list\left[F_{i}^{MFP}\right]$16:**for **i=1,...,5**do**17:    $F^{\prime }\leftarrow$ DownSampling($F_{0}$ if i==1 else $F_{i-1}^{ENC}$, 1/2)18:    $F_{i}^{\prime MFP}\leftarrow$ ChannelAttention($\theta_{channel}^{Attention},F_{i}^{MFP}$))19:    $F_{i}^{ENC}\leftarrow$ DSPC($\theta^{DSPC}$, Concatenate($F^{\prime },F_{i}^{\prime MFP}$))    {See Algorithm 3 for details}20:**end for**21:**return **$list[F_{i}^{ENC}]$22: 23:**Module**    DEC    Decoder24:**input **$F_{0},list\left[F_{i}^{ENC}\right]$25:**for **i=5,...,1**do**26:    $F^{\prime }\leftarrow$ UpSampling($F_{5}^{ENC}$ if i==5 else $F_{i+1}^{DEC}$, 2)27:    $F^{\prime \prime }\leftarrow$ ChannelAttention($\theta_{channel}^{Attention},F_{0}$ if i==1 else $F_{i-1}^{ENC}$))28:    $F_{i}^{DEC}\leftarrow$ DSPC($\theta^{DSPC}$, Concatenate($F^{\prime },F^{\prime \prime }$))29:**end for**30:**return **$F_{5}^{DEC}$31: 32:**Module**    Output Layer33:**input **$F_{5}^{DEC}$34:P← Discrete(Sigmoid(Conv($\theta_{output},F_{5}^{DEC}$)), 0.5)35:**return** *P*


**Algorithm 2** MFP-*i*: The *i*th Multiple Feature Pyramid Path**Require:** Input Feature Maps: $F_{input}\left[B,C,H,W,D\right]$ *B: Batch size, C: Channel (MRI sequences), H: Height, W: Width, D: Depth*      Initialized Weight Dictionary: $\Theta \{\theta_{i,1}^{DSPC},\ldots ,\theta_{i,i}^{DSPC}\}$ **Ensure:** the *i*th Feature Pyramid: $F_{i}^{MFP}$
1:$F_{i,0}\leftarrow$ TrilinearInterpolation($F_{input},1/2^{i}$)2:**for **j=1,…,i**do**3:    $F_{i,j}\leftarrow DSPC_{ij}\left(\theta_{i,j}^{DSPC},F_{i,j-1}\right)$    {See Algorithm 3 for details}4:**end for**5:**return **$F_{i,i}$ as $F_{i}^{MFP}$


**Algorithm 3** DSPC Module: Depth-wise separable convolution with hybrid attention module**Require:** Intermediate Feature Map: $F_{in}\left[B,C,H,W,D\right]$      Initialized Weight Dictionary: $\Theta \{\theta_{depth}^{Conv},\theta_{point}^{Conv},\theta_{s}^{Att},\theta_{c}^{Att}\}$ **Ensure:** DSPC’s Output Feature Map: $F_{out}v\left[B,C^{\prime },H,W,D\right]$
1:$F^{\prime }\leftarrow$ GroupNorm(DepthwiseConv($\theta_{depth}^{Conv},F_{in}$))2:$F^{\prime }\leftarrow$ ELU(SpatialAttention($\theta_{s}^{Att},F^{\prime }$))3:$F^{\prime }\leftarrow$ GroupNorm(PointwiseConv($\theta_{point}^{Conv},F^{\prime }$))4:$F^{\prime }\leftarrow$ Add($F_{in}$, ELU($F^{\prime }$))5:$F^{\prime }\leftarrow$ ChannelAttention($\theta_{c}^{Att},F^{\prime }$)4:**return** $F^{\prime }$ as $F_{out}$


### 3.2. Multiple Feature Pyramid Paths

**Figure 3:**
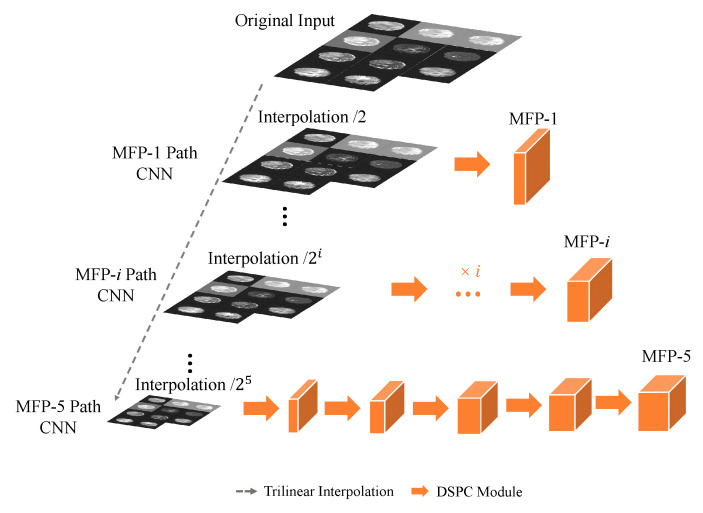
Multiple Feature Pyramid (MFP) paths.

As depicted in Figure 3, the MFP paths in N-LiNet adopt a multi-path CNN approach to construct a feature pyramid, where each MFP path is denoted as MFP-*i*. At the onset of each path, the input data undergo down-sampling using trilinear interpolation, reducing the resolution by a power of 2 corresponding to the path index (*i*). This down-sampling technique ensures a smooth and continuous approximation of the input data, effectively preserving the spatial relationships and structural information inherent in the original image.

Following the down-sampling step, the data proceed through a series of *i* sets of DSPC basic convolutional modules. These modules efficiently capture and encode local spatial patterns, thereby enhancing the discriminating capabilities of the extracted features. Through this hierarchical processing, the MFP-*i* path generates a feature pyramid that encompasses multi-scale information, enabling the network to capture both fine-grained details and high-level contextual information.

Subsequently, the resulting feature pyramid is fed into the encoder component of N-LiNet for further processing. The details of MFP-*i* are outlined in Algorithm 2.

By leveraging the MFP paths and their associated feature pyramids, N-LiNet effectively integrates multi-scale information and facilitates the extraction of informative features for accurate and robust analysis. This integration of multi-scale features from the MFP paths into the encoder allows for the model to leverage a rich representation that encompasses both local details and global contextual information.

### 3.3. Depth-Wise Separable Convolution with Hybrid Attention module

**Figure 4:**
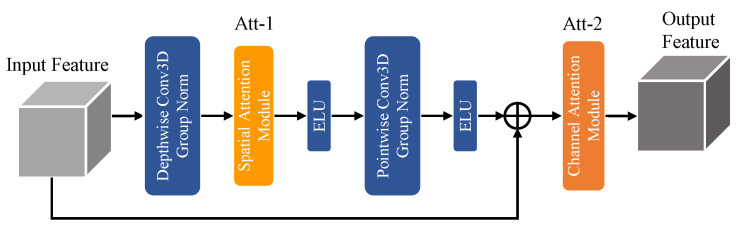
DSPC Module.

To achieve harmonious synergy between exceptional segmentation performance and reducing model parameter size and computational complexity, we propose the DSPC module depicted in Figure 4. This module combines the principles of depth-wise separable convolution [19] and fusion attention mechanisms.

In practical applications, we observed that simply tailing various attention mechanisms after depth-wise separable convolution did not yield the desired results. This limitation arises from the fact that depth-wise convolution extracts features solely in the spatial dimensions of the feature maps, while point-wise convolution focuses on channel-wise feature extraction. To address this challenge, we devised a novel module that follows a sequential order of depth-wise convolution, spatial attention, point-wise convolution, and channel attention. By integrating these components in a specific order, we capitalized on the strengths of each operation. The depth-wise convolution captures spatial details efficiently, while the spatial attention mechanism enhances the discriminating power of the extracted features by highlighting salient spatial regions. The subsequent point-wise convolution extracts channel-wise features, and the channel attention mechanism further refines the feature representation by emphasizing important channels and suppressing irrelevant ones. Algorithm 3 offers the implementation details and the design flow of the DSPC module.

This novel design enables the extraction of both spatial and channel information while effectively reducing the parameter size and computational complexity of the model. In the N-LiNet architecture, incorporating the DSPC module results in a reduction in the parameter size by a factor of 0.24 and a decrease in multiply accumulate computations (MACs) by a factor of 0.52 compared to the original configuration.

### 3.4. Horizontal Skip Connection with Channel Attention

The skip connections in N-LiNet facilitate the horizontal propagation of feature maps between corresponding levels in the MFP paths, encoder, and decoder. This interconnection plays a crucial role in the network’s ability to capture both local and global contextual information, as well as to fuse features from different scales. Incorporating attention mechanisms within skip connections is a commonly used optimization technique [29]. However, conventional attention gates may not always yield the most suitable results. This is because attention gates typically focus on capturing spatial dependencies but may not adequately capture channel-wise dependencies, which are crucial for feature representation.

Through experimental investigations, we discovered that the most effective attention mechanism to be integrated into skip connections is the channel attention mechanism. This choice is driven by channel attention’s ability to effectively capture the inter-dependencies between channels within the feature maps, enabling the model to emphasize informative channels while suppressing less relevant ones. By incorporating channel attention within skip connections, N-LiNet can dynamically adapt its feature representation, enhancing its ability to capture discriminating information and improving its segmentation performance.

### 3.5. Combo Loss with Weighted Cross-Entropy and Dice Loss

In the task of brain tumor image segmentation, three specific tumor sub-regions need to be considered: the Enhancing Tumor (ET), the Tumor Core (TC), and the Whole Tumor (WT). The ET typically represents the active portion of the tumor and may consist of highly invasive tumor cells. The TC describes the main part of the tumor, which often corresponds to the region that is surgically removed and includes the ET as well as the Necrotic Region (NCR). The WT encompasses the overall extent of the tumor, including both the TC and the Edema (ED) region. Two types of label imbalance issues need to be addressed. Firstly, there is an imbalance among different sub-regions within the brain tumor. Secondly, there is an imbalance between the tumor region and the background, where the background is typically much larger than the tumor region.

To tackle these imbalance problems, N-LiNet employs a combo loss function of weighted cross-entropy loss [22] and Dice loss [23], formulated as follows:

(1) $$\begin{array}{cc} \; & L_{Combo}=L_{Dice}+L_{WCE}, \end{array}$$

(2) $$\begin{array}{cc} \; & L_{Dice}=1-\frac{2}{\left|K\right|}\sum_{k\in K}\frac{\sum_{i\in N}y_{i}p_{i}^{k}}{\sum_{i\in N}y_{i}+\sum_{i\in N}p_{i}^{k}}, \end{array}$$

(3) $$\begin{array}{cc} \; & L_{WCE}=-\frac{1}{\left|N\right|}\sum_{i\in N}\sum_{k\in K}w^{k}y_{i}ln(p_{i}^{k}), \end{array}$$

where $p_{i}^{k}$ represents the probability of predicting the *i*th voxel as belonging to tumor sub-region *k*, while $y_{i}$ represents the corresponding ground truth label. The set of all voxels in the brain tumor image is denoted as *N*, and the set of tumor sub-regions is denoted as *K*. Function ln denotes the natural logarithm. $w^{k}$ represents the weight assigned to tumor sub-region *k*, and its formula is

(4) $$w^{k}=\frac{\left|WT\right|}{\left|k\right|},$$

where $\left|k\right|$ represents the voxel count in the tumor sub-region, while $\left|WT\right|$ represents the voxel count in the WT region. In this study, the voxel count of the tumor sub-region is used instead of that of the segmentation label to calculate the weight because tumor sub-regions are composed of nested segmentation labels, allowing for a more appropriate balancing of the weights assigned to each tumor sub-region.

## 4. Experiment Settings

### 4.1. Datasets

This study evaluates the segmentation performance using four different brain tumor segmentation datasets: UCSF-PDGM, BraTS2021, BraTS2019, and MSD’s Task-01.

**Figure 5:**
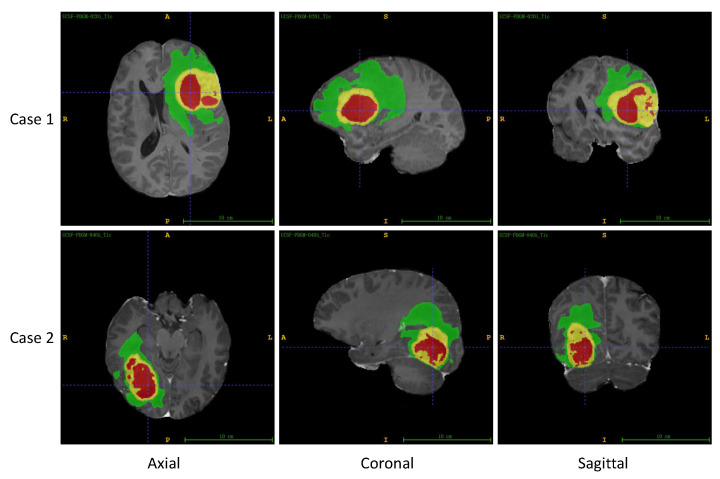
Two sets of MRI images and corresponding segmentation labels from UCSF-PDGM dataset, including axial, coronal, and sagittal views. In this figure, ET is labeled yellow, TC is the union of yellow and red, and WT includes all the colored (yellow, red, and green) labels.

The UCSF-PDGM dataset is a publicly available dataset released by the University of California, San Francisco (UCSF) [33]. It comprises MRI images of 501 patients with histopathologically confirmed diffuse glioma. Each patient has skull-stripped co-registered 3D images from 11 different MRI sequences, along with three-compartment tumor segmentation. Figure 5 showcases two sets of representative MRI images and their corresponding segmentation labels obtained from the UCSF-PDGM dataset. The images are viewed from axial, coronal, and sagittal perspectives.

The BraTS dataset series is part of the Brain Tumor Segmentation Challenge and is a publicly available dataset [34]. It consists of multi-modal MRI image data from brain tumor patients collected from multiple institutions. The BraTS 2019 version provides 335 publicly available sets of brain tumor MRI images, while the BraTS 2021 version offers 1251 sets. Each patient’s images include T1-weighted, T1-weighted contrast-enhanced, T2-weighted, and FLAIR sequences. Additionally, the dataset provides expert-labeled tumor segmentation masks.

The MSD dataset is a publicly available medical image segmentation dataset aimed at fostering research and comparison of medical image segmentation algorithms [35]. It comprises medical image data from ten different tasks, each focusing on a distinct organ or pathology. Task-01 within the MSD dataset specifically addresses brain tumor segmentation. This task provides multi-modal MRI image data from patients with High-Grade Gliomas (HGG) and Low-Grade Gliomas (LGG), totaling 484 sets of images. The MRI sequences of this task are the same as those of the BraTS dataset.

Public datasets provide ground truth annotations for brain tumor segmentation, which have been expertly labeled by medical professionals. The annotations specifically delineate the enhanced tumor region (ET), the necrotic and non-enhancing tumor core region (NCR), and the edema region (ED). The ET and NCR regions collectively form the tumor core region (TC), while the TC and ED regions combined constitute the whole tumor region (WT).

The aforementioned datasets were divided into training, validation, and testing sets in a ratio of 7:1:2, respectively. The training set was subjected to the following data preprocessing techniques: first, *z*-score normalization was applied to standardize the training data and reduce scale differences between samples. Subsequently, random flipping, random rotation, and random cropping were performed to enhance the diversity of the training samples while ensuring a unified image resolution of 192×192×128. Lastly, Gaussian noise was added to the images to simulate real-world noise and variations in image quality, facilitating the model’s adaptation to different noise conditions.

### 4.2. Evaluation Metrics

This study evaluates the segmentation performance of different methods on the test set using three commonly used metrics for brain tumor segmentation tasks: mean Intersection over Union (mIoU), Dice Similarity Coefficient (DICE), and 95th percentile of Hausdorff Distance (HD95).

The mIoU quantifies the similarity between predicted and ground truth regions. It is calculated as the ratio of the intersection to the union of the predicted and true regions:

(5) mIoU=TP/(TP+FP+FN),

where TP represents the number of true positive samples, FP represents the number of false positive samples, and FN represents the number of false negative samples. Each voxel is considered as an individual sample. The closer the mIoU value is to 1, the greater the similarity between the predicted and true regions.

The DICE measures the overlap between the predicted and ground truth regions. It emphasizes the accuracy of positive samples in the segmentation result. The DICE is calculated using the following formula:

(6) DICE=2TP/(2TP+FP+FN).

where a higher DICE value indicates a higher degree of overlap between the predicted and true regions.

The Hausdorff Distance (HD) is a metric used to quantify the surface shape differences between the segmentation result and ground truth labels. It measures the maximum unidirectional surface distance between the predicted and true regions. The HD is calculated using the following formula:

(7) $$\begin{array}{cc} HD(P,Y) & =\max(h(P,Y),h(Y,P)), \end{array}$$

(8) $$\begin{array}{cc} h(P,Y) & =\max_{p\in P}\left(\min_{y\in Y}\left(\left|p-y\right|\right)\right), \end{array}$$

where h(P,Y) represents the maximum single-directional surface distance from the predicted region set *P* to the ground truth label set *Y*. Term $\left|p-y\right|$ denotes the Euclidean spatial distance between two points p and y. In practice, to mitigate the impact of outliers on the evaluation metrics, the 95th percentile of the surface distances denoted as HD95 is often used. A smaller HD95 value indicates a smaller shape difference between the segmentation result and ground truth labels.

### 4.3. Other Details

All experiments in this study were conducted under a unified environment configuration. The hardware platform consisted of an Intel Xeon Gold 6248R processor with 72 GB of memory and an NVIDIA A100 graphics card with 80 GB of GPU memory. The implementation and training of models were carried out using the PyTorch 2.0.1 deep learning framework and the Python 3.8 programming language. The CUDA 11.8 backend acceleration library was employed. The experimental procedures were executed on the Ubuntu 20.04 operating system. During model training, a batch size of 1 was employed due to the GPU memory constraint while the training epochs were set to 200. For better brain tumor segmentation performance [52] and fair comparison, we chose AdamW [53] as the optimizer, with an initial learning rate of $1\times 10^{-4}$ and a weight decay coefficient of $1\times 10^{-5}$.

## 5. Experimental Evaluation and Discussion

For the collection tables in this section, individual metrics for ET, TC, and WT tumor subregions, as well as their respective mean values were recorded for each method. The representation of the metrics was rounded to two decimal places.

### 5.1. Segmentation Performance Comparison

**Figure 6:**
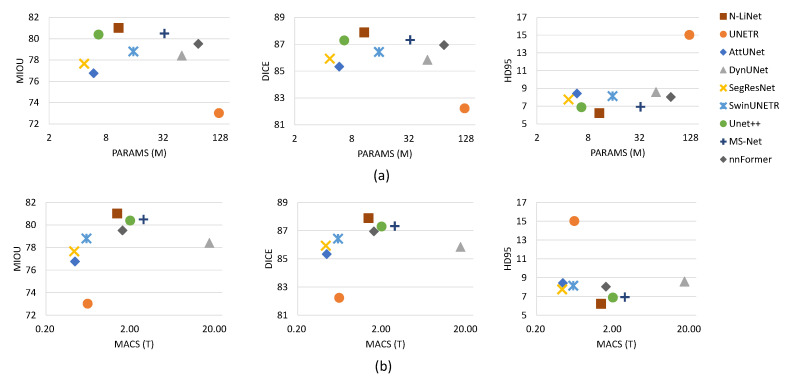
Comparison on the UCSF-PDGM dataset with (**a**) parameter size and (**b**) computational complexity comparison over mIoU, Dice, and HD95.

In this section, a comprehensive comparison was conducted between N-LiNet and other state-of-the-art segmentation models on four brain tumor datasets. The segmentation metrics were collected, and the results are presented in Table 1, Table 2, Table 3, and Table 4, respectively. In Figure 6, the parameter sizes and computational complexities of different segmentation methods are presented.

#### 5.1.1. Segmentation Metrics Comparison

For the UCSF-PDGM dataset, N-LiNet exhibits notable superiority over other models in terms of evaluation metrics. Specifically, for the mIoU metric, N-LiNet demonstrates the most substantial improvement compared to UNETR, with an average increase of 11.0%. In addition, the improvement relative to UNet++ is comparatively modest, with an average increase of 0.8%. Regarding the DICE metric, N-LiNet shows an average improvement of 6.9% compared to UNETR and an average improvement of 0.7% compared to UNet++. It is worth noting that N-LiNet demonstrates the most pronounced optimization in terms of the HD95 metric. Compared to UNETR, N-LiNet achieves a significant reduction of 58.7%, while compared to UNet++, it also achieves a reduction of 9.7%. There is only one special case for N-LiNet, which exhibits a slightly inferior performance compared to UNet++ in terms of HD95 for ET. On the remaining three datasets, N-LiNet exhibits similar superior segmentation performance, although the detailed results are not explicitly described here.

Regarding the relatively inconspicuous improvement in mIOU and Dice metrics compared to the substantial improvement in HD95, we acknowledge that the progress may appear less pronounced. However, it is important to note that even among other state-of-the-art models, the differences in mIOU and Dice metrics are not always significant. In this context, our method still demonstrates a clear improvement in these metrics compared to existing methods. The comparative experimental results mentioned above unequivocally showcase the outstanding segmentation performance of N-LiNet in brain tumor segmentation tasks. This highlights its remarkable superiority in terms of segmentation overlap, tumor completeness, and shape correspondence within the segmented results.

#### 5.1.2. Parameter Size and Computational Complexity Comparison

Without loss of generality, only the results of the UCSF-PDGM dataset are considered in the experiments later in this paper.

In Figure 6a, the left-to-right arrangement of the three scatter plots illustrates the corresponding distributions of parameter sizes and average segmentation metrics (mIoU, DICE, and HD95) for different methods. The vertical axis represents the values of segmentation metrics, while the horizontal axis represents parameter sizes, measured in millions. Similarly, in Figure 6b, the left-to-right arrangement of the three scatter plots illustrates the corresponding distributions of computational complexities and segmentation metrics. Computational complexity is represented in terms of the number of TeraFLOPs (trillions of floating-point operations) required for multiply-accumulate computations. To ensure clear visualization without overcrowding, a logarithmic scale with a base of 2 is employed for the horizontal axis in all subplots.

In comparison to UNet++, our method presents a trade-off between parameter size and segmentation performance. However, it is worth noting that UNet++ employs a highly complex nested and dense structure and relies on deep supervision techniques, making the training process significantly more complex and time-consuming. As demonstrated in Figure 6, although UNet++ has a relatively smaller parameter size, the computational complexity is noticeably higher, providing evidence for this observation. In contrast, our method utilizes a supervised training approach similar to U-Net, which is less computationally demanding. From the results depicted in Figure 6, it can be observed that N-LiNet achieves excellent brain tumor segmentation performance while maintaining low parameter size and computational complexity. This finding underscores the effectiveness of the proposed method in this study.

### 5.2. Performance Comparison of the DSPC Module

As illustrated in Figure 4, the DSPC module consists of two attention modules, referred to as Att_1 and Att_2. In this section, a series of comparative experiments were conducted by substituting different attention mechanisms at these two positions to investigate the impact of the DSPC module configuration on the performance of brain tumor segmentation. The experimental results are presented in Table 5, where the symbol “×” indicates the absence of an attention mechanism at that specific position. The term “Coor” denotes the utilization of coordinate attention, “SA” represents the incorporation of spatial attention, “CA” signifies the adoption of channel attention, and “CBAM” indicates the incorporation of CBAM attention.

We can observe that even by simply swapping the coordinate attention mechanism from position Att_1 to Att_2, the segmentation performance of the network can be improved to some extent. This highlights the importance of placing different attention mechanisms in suitable positions to fully leverage their performance advantages. Additionally, it is worth noting that although the combination of “SA” and “Coor” yields some benefits, the improvement is not significant and even falls short compared to the introduction of “CA” attention at position Att_2 alone. On the other hand, while using CBAM attention at the Att_2 position can achieve relatively good segmentation performance, it is inferior to the effect achieved by introducing “SA” at the Att_1 position and “CA” at the Att_2 position, i.e., the DSPC module.

Thus, our experimental results demonstrate that the proposed structure of the DSPC module, which involves integrating the hybrid attention module into the DSC module rather than simply concatenating them in sequence, allows us achievement of optimal segmentation performance.

### 5.3. Comparing Different Attention Mechanisms on Horizontal Skip Connections

Similarly, we conducted comparative experiments by adding different attention mechanisms to the skip connections in the horizontal direction. The experimental results summarized in Table 6 provide insights into the impact of different attention mechanisms on the performance of the network when applied to horizontal skip connections. In this section, we selected the N-LiNet model, which consists of only the lightweight DSC module without any additional attention mechanisms, as the “Basic” model. This choice was made to eliminate the potential influence of other attention mechanisms on the model’s segmentation performance.

Based on the experimental results, it is evident that both the coordinate attention and CBAM attention, which are theoretically expected to exhibit stronger performance, actually demonstrate inferior results compared to the individual spatial attention or channel attention mechanisms. Meanwhile, the channel attention mechanism plays the most prominent role in skip connections. This can be attributed to its ability to adaptively assign importance to different channels, facilitating improved feature representation and discrimination. By selectively emphasizing informative channels, the channel attention mechanism enhances the discriminating power of the network and contributes to more accurate tumor segmentation results.

Overall, these findings highlight the importance of selecting attention mechanisms that are well-suited to the characteristics of the segmentation task to achieve optimal performance.

### 5.4. Abalation Experiments

To evaluate the impact of different optimization methods proposed in this study on the performance of N-LiNet in brain tumor segmentation tasks, a series of ablation experiments were conducted, and the results are collected in Table 7. Concise but without loss of generality, we only experimented on the UCSF-PDGM dataset.

In Table 7, the “Basic” configuration represents the foundational 3D U-Net model structure, while “MFP” denotes the Multi-Feature Pyramid module. The “DSC” module corresponds to the standard f Separable Convolution, while “DSPC” represents the Depth-wise convolution, Spatial attention, Point-wise convolution, and the Channel attention module. Lastly, “HCA” signifies the Horizontal skip connections with Channel Attention modules.

By comparing the results of the first two rows in Table 7, we can validate the significant optimization of the MFP module on the segmentation performance. Specifically, the model achieves an average improvement of 3.4% in mIoU, 2.0% in DICE, and a reduction of 14.7% in HD95. However, from the results of the third row, it can be observed that the DSC module degrades the model’s segmentation performance to a large extent. The results from the fourth and fifth rows indicate that both HCA and DSPC effectively enhance the model’s segmentation performance. However, the combination of “MFP+HCA+DSC” does not outperform the sole “MFP” optimization method. Ultimately, the combination of “MFP+HCA+DSPC”, which corresponds to the proposed N-LiNet structure in this study, achieves the best segmentation performance overall. Compared to the “Basic” model, the N-LiNet model achieves an average increase of 3.5% in mIoU, 2.4% in DICE, and a reduction of 21.3% in HD95.

### 5.5. Combo Loss Function

Table 8 summarizes the performance of N-LiNet when trained with different loss functions. It provides insights into the effectiveness of DiceWCE Loss in comparison to the alternative loss functions commonly employed in medical image segmentation, such as Dice Loss and Dice-CE Loss. From the results, it is evident that DiceWCE Loss outperforms the other two loss functions across all three segmentation metrics.

### 5.6. Discussion

**Figure 7:**
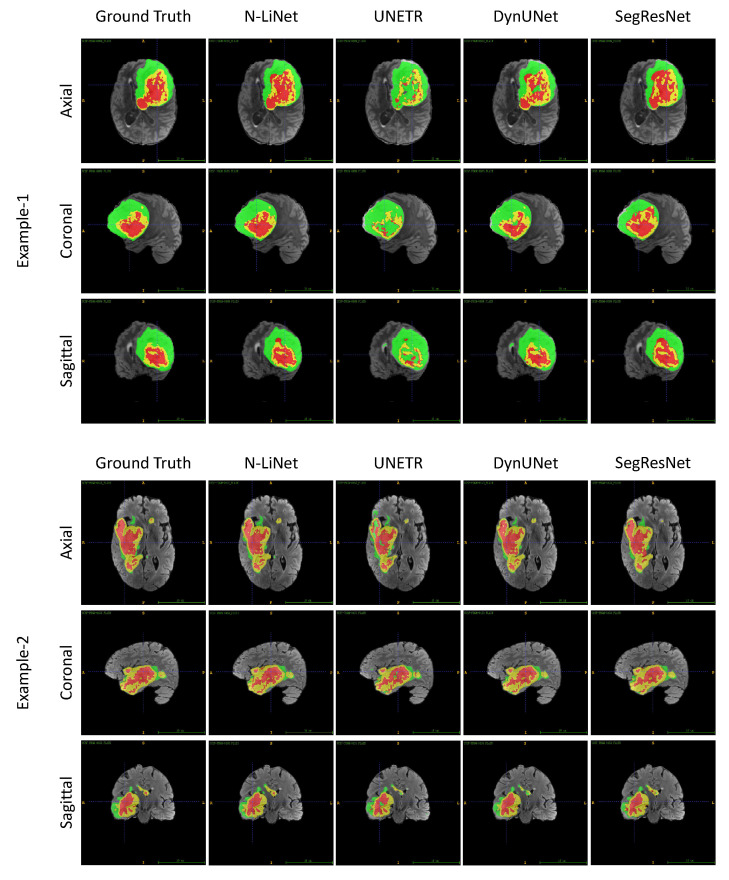
Comparing the segmentation results of different networks visually based on the two examples of UCSF-PDGM dataset from axial, coronal, and sagittal views, respectively. In this figure, ET regions are labeled with yellow color, TC regions are the union of yellow and red areas, and WT regions include all the three colored (yellow, red, and green) labels.

Based on the experimental results presented above, we not only validated the superior performance of N-LiNet compared to other state-of-the-art models in brain tumor segmentation, but also conducted comparative and ablation experiments to analyze the effectiveness of different optimization methods proposed in this study. Ultimately, we demonstrated the efficacy of these methods. Moving forward, we can further compare N-LiNet with other segmentation models visually. In Figure 7, two sets of segmentation examples are presented. In each set, from left to right, we show brain tumor MRI images with the corresponding ground truth labels and the predicted results from four different network models.

In Example 1, we can observe that the tumor region segmented by N-LiNet closely resembles ground truth labels. The large NCR region colored in red is accurately delineated, and the fragmented details of the central ET region colored in yellow are also captured precisely. In contrast, UNETR almost fails to correctly segment the ET region and the NCR region. DynUNet exhibits numerous false negatives and wrong labels in the central TC areas. SegResNet, on the other hand, successfully segments the WT region relatively well but shows incompleteness with the NCR region and loses significant details of the ET region.

For Example 2, the tumor exhibits a more irregular shape; however, N-LiNet is still able to accurately segment various regions of the tumor, with significantly fewer false positive results. Conversely, the UNETR model exhibits noticeable mislabeling in the ET and NCR regions and fails to accurately segment certain small tumor areas. DynNet and SegResNet, on the other hand, produce a higher number of false positive regions.

## 6. Conclusions

We propose a novel N-LiNet model for brain tumor segmentation tasks. N-LiNet combines the concepts of feature pyramids and U-Net, constructing an N-shaped neural network that incorporates multi-scale feature extraction pathways in addition to the traditional encoder–decoder structure. Furthermore, N-LiNet integrates various attention mechanisms into depth-wise separable convolution modules and skip connections, effectively improving segmentation performance while controlling model parameter size and computational complexity at a lower level. Additionally, this study designs a combo loss function of dice loss and weighted cross-entropy loss to address the issue of region imbalance in the tumors.

The choice of N-LiNet over U-Net and other architectures is supported by experimental results obtained from multiple publicly available datasets. These results demonstrate that the proposed N-LiNet model achieves superior performance in brain tumor segmentation compared to U-Net and other state-of-the-art architectures. Our evaluation metrics and visual analysis of the results indicate that N-LiNet has clear advantages in brain tumor segmentation. In particular, N-LiNet exhibits higher accuracy in segmenting tumor regions and excels in capturing intricate details within these regions. Compared to other mainstream models, such as U-Net and its variants, N-LiNet also demonstrates relatively good performance in handling tumors with irregular shapes, resulting in a lower false positive rate. These findings highlight the exceptional performance and potential of N-LiNet in the realm of brain tumor segmentation. Additionally, a key consideration in choosing N-LiNet is its favorable balance between model complexity and computational efficiency. N-LiNet achieves state-of-the-art performance while maintaining a manageable model size and computational requirements, making it practical for real-world deployment and clinical applications.

In future work, we will further explore how the N-shaped neural network can achieve model generalization across different datasets, thereby reducing the need for retraining and fine-tuning on specific datasets.

## Figures and Tables

**Table 1:** Segmentation performance on the UCSF-PDGM dataset.

Models	mIoU (%) ↑				DICE (%) ↑				HD95 ↓			
	**ET**	**TC**	**WT**	**Mean**	**ET**	**TC**	**WT**	**Mean**	**ET**	**TC**	**WT**	**Mean**
UNETR [30]	70.93	70.80	77.33	73.02	81.02	79.58	86.08	82.23	8.65	12.93	23.48	15.02
U-Net [24]	71.17	76.41	78.25	75.27	82.45	83.21	86.59	84.08	6.79	8.29	14.59	9.89
AttUNet [29]	73.25	76.50	80.54	76.76	83.47	83.98	88.55	85.33	4.84	8.64	11.78	8.42
DynUNet [27]	74.34	79.44	81.46	78.41	83.39	85.27	88.84	85.84	4.91	7.88	12.94	8.58
SegResNet [26]	73.33	78.55	81.09	77.66	83.59	85.31	88.88	85.93	5.88	7.00	10.37	7.75
SwinUNETR [28]	75.31	79.33	81.77	78.80	84.48	85.76	89.06	86.43	4.87	7.32	12.19	8.13
nnFormer [32]	76.44	80.15	81.97	79.52	85.19	86.07	89.57	86.94	4.81	7.16	12.11	8.03
UNet++ [25]	77.23	82.02	81.94	80.39	85.61	87.16	89.09	87.29	3.57	6.48	10.57	6.88
MS-Net [31]	76.57	81.82	83.09	80.49	85.19	87.18	89.58	87.32	5.35	5.96	9.46	6.92
N-LiNet	77.36	82.11	83.59	81.02	85.78	87.49	90.38	87.89	4.80	4.99	8.83	6.21

↑ denotes that higher values of the metrics are better, and ↓ denotes that lower values are better.

**Table 2:** Segmentation performance on the BRATS 2021 dataset.

Models	mIoU (%) ↑				DICE (%) ↑				HD95 ↓			
	**ET**	**TC**	**WT**	**Mean**	**ET**	**TC**	**WT**	**Mean**	**ET**	**TC**	**WT**	**Mean**
UNETR [30]	73.62	76.41	81.62	77.22	82.49	84.67	89.02	85.39	8.78	10.22	14.42	11.14
AttUNet [29]	75.78	80.58	84.41	80.26	84.05	86.89	90.98	87.31	5.30	6.57	8.99	6.95
U-Net [24]	77.39	83.27	85.43	82.03	85.38	89.16	91.59	88.71	4.92	5.31	7.94	6.06
DynUNet [27]	77.65	83.35	85.53	82.18	85.52	89.29	91.60	88.80	4.82	5.61	7.88	6.10
SegResNet [26]	78.00	83.75	85.44	82.39	86.12	89.57	91.65	89.11	5.42	5.79	6.11	5.78
SwinUNETR [28]	78.50	83.57	85.87	82.65	86.12	89.26	91.75	89.04	4.96	5.32	7.19	5.82
nnFormer [32]	78.69	83.72	85.93	82.78	86.22	89.51	91.84	89.19	4.83	5.29	7.08	5.74
UNet++ [25]	78.40	84.19	85.82	82.80	86.15	89.85	91.82	89.27	4.98	5.37	7.51	5.95
MS-Net [31]	78.84	84.33	86.41	83.19	86.25	89.79	91.99	89.34	5.03	5.63	6.49	5.71
N-LiNet	79.80	85.78	87.10	84.23	87.17	91.13	92.60	90.30	4.00	4.48	6.03	4.84

↑ denotes that higher values of the metrics are better, and ↓ denotes that lower values are better.

**Table 3:** Segmentation performance on the BRATS 2019 dataset.

Models	mIoU (%) ↑				DICE (%) ↑				HD95 ↓			
	**ET**	**TC**	**WT**	**Mean**	**ET**	**TC**	**WT**	**Mean**	**ET**	**TC**	**WT**	**Mean**
U-Net [24]	62.56	68.02	73.98	68.18	73.61	77.79	83.07	78.15	8.79	10.29	16.59	11.89
AttUNet [29]	65.36	62.24	78.78	68.79	76.71	74.20	87.64	79.52	7.21	14.24	9.60	10.35
UNETR [30]	68.19	62.60	77.65	69.48	78.34	74.59	86.69	79.87	9.33	17.08	14.95	13.79
DynUNet [27]	70.01	73.31	79.84	74.39	79.70	82.54	88.24	83.49	5.97	9.63	19.27	11.62
SwinUNETR [28]	72.06	73.10	81.85	75.67	81.28	82.39	89.50	84.39	5.49	8.67	16.05	10.07
SegResNet [26]	69.19	77.10	80.91	75.73	79.59	85.20	89.00	84.60	7.12	6.88	7.22	7.07
nnFormer [32]	71.15	74.26	81.93	75.78	81.33	82.56	89.67	84.52	6.55	8.32	13.42	9.43
UNet++ [25]	70.95	76.01	81.72	76.23	80.33	84.55	89.54	84.81	7.10	9.35	12.59	9.68
MS-Net [31]	71.76	75.25	82.12	76.38	81.37	83.72	89.84	84.98	5.95	8.59	9.57	8.04
N-LiNet	72.62	77.41	83.54	77.86	81.91	86.16	90.65	86.24	5.91	6.21	7.58	6.57

↑ denotes that higher values of the metrics are better, and ↓ denotes that lower values are better.

**Table 4:** Segmentation performance on the MSD Task 01 dataset.

Models	mIoU (%) ↑				DICE (%) ↑				HD95 ↓			
	**ET**	**TC**	**WT**	**Mean**	**ET**	**TC**	**WT**	**Mean**	**ET**	**TC**	**WT**	**Mean**
UNETR [30]	67.76	64.10	76.78	69.55	78.62	76.34	86.28	80.41	10.06	14.17	19.57	14.60
U-Net [24]	68.01	69.71	78.77	72.17	78.72	80.17	87.02	81.93	7.01	10.96	12.97	10.31
AttUNet [29]	69.66	68.83	78.92	72.47	79.86	80.23	87.83	82.64	5.62	9.69	12.66	9.33
DynUNet [27]	70.12	71.31	79.98	73.80	80.80	82.05	88.45	83.76	5.50	9.94	13.44	9.63
UNet++ [25]	70.76	72.46	80.39	74.54	81.31	82.85	88.73	84.30	4.99	10.02	11.58	8.86
SegResNet [26]	69.82	72.79	81.01	74.54	80.48	83.02	89.17	84.23	5.22	7.93	9.14	7.43
SwinUNETR [28]	71.35	72.99	81.08	75.14	81.83	82.52	89.10	84.48	4.81	7.78	8.80	7.13
nnFormer [32]	71.36	73.03	81.15	75.18	81.94	82.65	89.27	84.62	4.79	7.73	8.75	7.09
MS-Net [31]	71.12	73.19	81.28	75.20	81.78	82.71	89.53	84.67	4.92	8.21	7.98	7.04
N-LiNet	71.99	72.97	82.23	75.73	82.46	82.98	89.89	85.11	4.53	7.70	7.87	6.70

↑ denotes that higher values of the metrics are better, and ↓ denotes that lower values are better.

**Table 5:** Performance comparison of the DSPC module on the UCSF-PDGM dataset.

Att_1	Att_2	mIoU (%) ↑				DICE (%) ↑				HD95 ↓			
		**ET**	**TC**	**WT**	**Mean**	**ET**	**TC**	**WT**	**Mean**	**ET**	**TC**	**WT**	**Mean**
×	×	72.49	75.23	81.29	76.33	82.49	83.05	88.79	84.78	6.09	8.48	11.63	8.73
Coor [37]	×	75.55	79.21	82.86	79.20	84.27	85.15	89.52	86.31	4.65	6.11	9.36	6.71
×	Coor [37]	75.99	81.13	82.77	79.96	85.03	86.95	90.09	87.36	5.32	6.27	7.88	6.49
SA [20]	Coor [37]	76.22	80.78	82.97	79.99	85.28	86.91	90.16	87.45	5.47	6.04	8.97	6.83
×	CA [21]	76.30	80.57	83.53	80.14	84.94	86.32	90.06	87.11	5.05	7.27	9.07	7.13
SA [20]	×	76.06	80.77	82.75	79.86	84.40	86.09	89.35	86.62	4.89	6.25	9.03	6.72
×	CBAM [36]	77.09	80.84	83.46	80.47	86.01	86.88	90.14	87.67	4.81	6.37	8.51	6.56
SA [20]	CA [21]	77.36	82.11	83.59	81.02	85.78	87.49	90.38	87.89	4.80	4.99	8.83	6.21

↑ denotes that higher values of the metrics are better, and ↓ denotes that lower values are better.

**Table 6:** Comparing different attention mechanisms on horizontal skip connections on the UCSF-PDGM dataset.

Configs	mIoU (%) ↑				DICE (%) ↑				HD95 ↓			
	**ET**	**TC**	**WT**	**Mean**	**ET**	**TC**	**WT**	**Mean**	**ET**	**TC**	**WT**	**Mean**
Basic	72.49	75.23	81.29	76.33	82.49	83.05	88.79	84.78	6.09	8.48	11.63	8.73
+Coor [37]	74.34	78.70	80.29	77.77	83.44	84.84	87.88	85.38	5.78	6.56	10.61	7.65
+CBAM [36]	73.97	79.05	80.83	77.95	83.30	85.22	88.23	85.58	5.94	6.61	9.41	7.32
+SA [20]	74.60	79.62	81.24	78.48	83.54	85.42	88.39	85.78	6.11	6.79	8.65	7.18
+CA [21]	74.70	79.52	81.09	78.44	84.06	85.78	88.50	86.11	5.38	6.52	9.50	7.13

↑ denotes that higher values of the metrics are better, and ↓ denotes that lower values are better.

**Table 7:** Ablation experiments on the UCSF-PDGM dataset.

Configs	mIoU (%) ↑				DICE (%) ↑				HD95 ↓			
	**ET**	**TC**	**WT**	**Mean**	**ET**	**TC**	**WT**	**Mean**	**ET**	**TC**	**WT**	**Mean**
Basic	74.18	79.41	81.26	78.28	83.54	85.32	88.69	85.85	4.79	6.29	12.59	7.89
+MFP	77.59	82.42	82.91	80.97	85.61	87.42	89.77	87.60	5.75	6.20	8.26	6.73
+MFP+DSC	72.49	75.23	81.29	76.33	82.49	83.05	88.79	84.78	6.09	8.48	11.63	8.73
+MFP+HCA+DSC	74.70	79.52	81.09	78.44	84.06	85.78	88.50	86.11	5.38	6.52	9.50	7.13
+MFP+HCA+DSPC	77.36	82.11	83.59	81.02	85.78	87.49	90.38	87.89	4.80	4.99	8.83	6.21

↑ denotes that higher values of the metrics are better, and ↓ denotes that lower values are better.

**Table 8:** Metrics of N-LiNet with different loss functions on the UCSF-PDGM dataset.

Loss	mIoU (%) ↑				DICE (%) ↑				HD95 ↓			
	**ET**	**TC**	**WT**	**Mean**	**ET**	**TC**	**WT**	**Mean**	**ET**	**TC**	**WT**	**Mean**
Dice	76.27	80.72	81.95	79.65	84.09	85.77	88.38	86.08	5.93	8.25	9.47	7.88
DiceCE	77.21	81.32	82.97	80.50	85.65	86.99	89.59	87.41	5.23	7.30	8.22	6.92
DiceWCE	77.36	82.11	83.59	81.02	85.78	87.49	90.38	87.89	4.80	4.99	8.83	6.21

↑ denotes that higher values of the metrics are better, and ↓ denotes that lower values are better.

## Data Availability

Data are contained within the article.
